# Improving the Accuracy of Predictive 2D-LC Optimization Strategies: Incorporation of Simulated Elution Profiles to Account for Injection Band Broadening in Online Comprehensive Two-Dimensional Liquid Chromatography

**DOI:** 10.1021/acs.analchem.4c00491

**Published:** 2024-04-09

**Authors:** Magriet Muller, Tyler Brau, Thomas Lauer, Dwight Stoll, André de Villiers

**Affiliations:** † Department of Chemistry and Polymer Science, University of Stellenbosch, Private Bag X1, Matieland, Stellenbosch 7602, South Africa; ‡ Department of Chemistry, Gustavus Adolphus College, 800 West College Avenue, Saint Peter, Minnesota 56082, United States

## Abstract

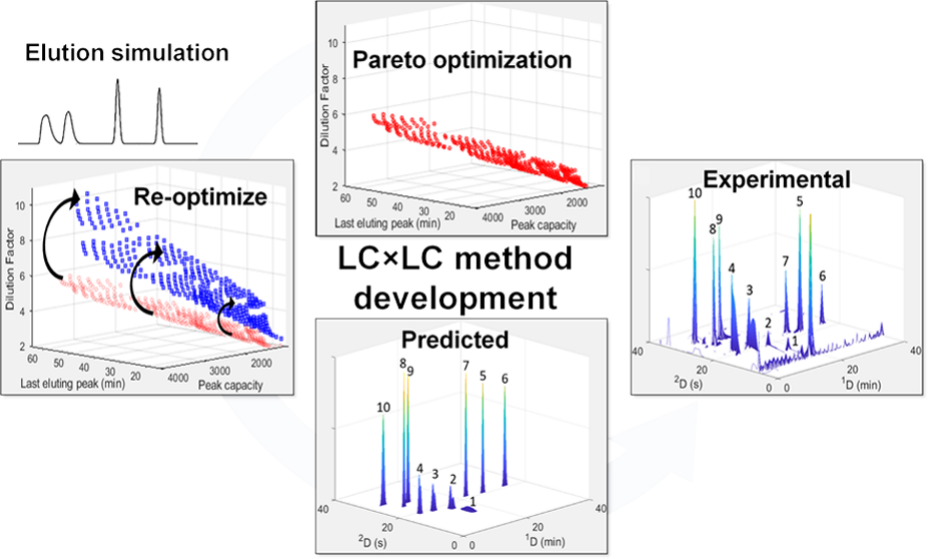

Method development in online comprehensive two-dimensional liquid chromatography (LC × LC) requires the selection of a large number of experimental parameters. The complexity of this process has led to several computer-based LC × LC optimization algorithms being developed to facilitate LC × LC method development. One particularly relevant challenge for predictive optimization software is to accurately model the effect of second dimension (^2^D) injection band broadening under sample solvent mismatch and/or sample volume overload conditions. We report a novel methodology that combines a chromatographic numerical simulation model capable of predicting elution profiles of analytes under conditions where peak distortion occurs with a predictive multiparameter Pareto optimization approach for online LC × LC. Preliminary method optimization is performed using a theoretical model to predict ^2^D injection profiles, and optimal experimental configurations obtained from the Pareto fronts are then subjected to further optimization using the simulation model. This approach drastically reduces the number of simulations and therefore the computational demand. We show that the optimal experimental conditions obtained in this manner are similar to those obtained using a complete optimization using only the simulation model. Online HILIC × RP-LC separation of phenolic compounds was used to compare experimental data to simulated two- and three-dimensional contour plots. The main advantage of the proposed approach is the ability to predict the formation of split or deformed peaks in the ^2^D, a significant benefit in online LC × LC method optimization, especially for separation combinations with mismatched mobile phases. A further benefit is that simulated elution profiles can be used for the visualization of predicted two-dimensional chromatograms for method selection.

In recent years, comprehensive two-dimensional liquid chromatography (LC × LC) has become a well-established separation technique for the analysis of complex samples because of its capability of exceeding the separation performance of one-dimensional LC (1D-LC). However, the enhanced separation power of LC × LC comes at the cost of more complex method development, which involves the consideration of a much larger number of experimental variables. General guidelines^1,2^ and stepwise optimization schemes^3−6^ are useful for manual or trial-and-error LC × LC method development. However, due to the large number of experimental parameters in two-dimensional LC (2D-LC), such optimization protocols generally require preselection of important parameters, thereby limiting their scope.

More complete method optimization approaches based on multiobjective or Pareto optimization have been proposed for method development in comprehensive multidimensional LC.^7−13^ These methodologies generally use algorithms to calculate the performance of separations that would result from all possible combinations of experimental parameters being optimized, within user-specified ranges. Computerization of these approaches allows for a large number of parameters to be simultaneously optimized, and routinely more than 10,000 combinations of experimental parameters are considered during optimization. The output of these methods is typically presented in the form of Pareto fronts, which contain optimum conditions for the specified optimization objectives (peak capacity, analysis time, dilution factor, resolution, etc.), thereby allowing the analyst to investigate and select between optimal experimental conditions for a given application.^7^ Pareto optimality algorithms use theoretical models that describe the relationships between experimental variables to calculate performance descriptors such as peak capacity and resolution. The ultimate utility of such algorithms, therefore, depends on the accuracy of the theoretical equations used and their ability to model experimental configurations.

In online LC × LC, one critical aspect that must be accounted for during method optimization is injection band broadening in the ^2^D, since this can drastically affect performance, especially when two separation modes with mismatched mobile phases, such as hydrophilic interaction chromatography (HILIC) and reversed-phase liquid chromatography (RP-LC), are employed. Several theoretical equations have been used to model injection band broadening in high-performance liquid chromatography (HPLC).^10,14−16^ However, these have been shown to be inaccurate when faced with modeling band broadening under solvent mismatch and injection volume overload conditions such as encountered in online LC × LC, where severe peak deformation often tends to occur.^13^

Stoll and co-workers^17,18^ recently developed an algorithm to simulate the elution profiles of analytes in HPLC, which has been shown to accurately model peak profiles affected by injection band broadening. This simulation model is based on the Craig counter-current distribution model and incorporates the highly asymmetrical injection profiles obtained from LC × LC valves to increase the simulation accuracy. In addition, the model also accounts for partial loop filling, which is commonly used in 2D-LC. Compared to theoretical equations used to model the degree of band broadening under certain experimental conditions, the simulation model provides additional information in terms of peak profiles, which may be a particularly valuable attribute in LC × LC method development. From the perspective of incorporation into LC × LC method development software, though, the main disadvantage of the simulation model is that the calculation of elution profiles for each analyte is computationally expensive: while theoretical injection equations require one calculation per analyte, the simulation algorithm performs a minimum of one calculation per theoretical plate of the column being used for each analyte. The time required to perform one simulation depends on several factors, but, on a normal desktop computer, 1000 simulations currently take approximately 1 h. Thus, using the simulation model as part of a Pareto optimality approach to LC × LC method development, where typically >10,000 conditions are evaluated per optimization, is currently not viable.

In this article, we propose a methodology to incorporate the injection simulation algorithm into a predictive LC × LC method optimization program as a means, first of all, to improve the accuracy of predictions for especially weakly retained analytes in the ^2^D under typical online LC × LC conditions. A second benefit of this methodology is that the availability of simulated elution profiles allows for the visualization of predicted two-dimensional chromatograms, which is a highly desirable feature for method selection. Note that the goal of this work is not to validate the simulation model or the Pareto optimality approach, since this has been done in previous work,^13,17,18^ but rather to explore the value added by the incorporation of the injection simulation model to LC × LC method development performed using a computerized optimization algorithm. The methodology is demonstrated for the online HILIC × RP-LC separation of phenolic standards, and experimental results are used to verify the accuracy of the predictions.

## Methodology

The predictive kinetic optimization approach used in the present work has been described previously,^13^ and will only be covered briefly. For the most accurate predictions, the program requires the user to provide input data for the analytes of interest (plate height and retention parameters) and column properties (porosity, flow resistance, and maximum operating pressure), as well as ranges within which column dimensions (length, diameter, and particle size) and experimental parameters (flow rates, sampling times, etc.) should be varied during optimization. Examples of all of the input parameters used in the present study are provided in Tables S1 and S2 in the Supporting Information (SI). Furthermore, suitable retention models for each dimension, an injection model, and solvents must be selected and instrumental restrictions (maximum operating pressure in both dimensions and maximum loop volume) specified. For this study, the adsorption (eq 1) and linear solvent strength (eq 2) models were used to model retention in HILIC and RP-LC, respectively.
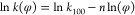
1
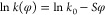
2Here, *k*(φ) is the retention factor (*k*) at a given solvent composition (φ), *k*_0_ and *k*_100_ are the extrapolated retention factors at 0 and 100% strong solvent, respectively, *n* is the number of strong solvent molecules displaced by a solute, and -*S* is the slope of a plot of the natural logarithm of the retention factor as a function of φ.

Equation 3 was used to calculate σ^2^*_inj_*, the variance of the sample plug due to the injection process^11,20^
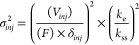
3where *V*_*inj*_ is the sample volume, *F* is the flow rate, δ_*inj*_^2^ is a parameter related to the injection process (δ_*inj*_^2^ = 8 was used in this work^19^), *k*_e_ is the local retention factor of the analyte at the column exit, and *k*_ss_ is the analyte retention factor in the sample solvent. In the ^2^D, the sample solvent consists of the ^1^D elution solvent, as well as any diluent added to the ^1^D effluent prior to entering the ^2^D column. Equation 3 is referred to as the ‘theoretical injection equation’ throughout the manuscript. While alternative equations exist for the determination of σ_*inj*_^2^^15^ and are incorporated in our program as options, eq 3 is recommended when steep gradients and/or strong injection solvents are used.^11,20^

In the next step, the algorithm uses a set of theoretical equations to calculate the retention times^21^ and peak widths^22^ of the analytes in both dimensions, as well as the average peak capacity^23^ and dilution factor,^24^ for all of the possible combinations of experimental variables not exceeding the specified restrictions. (Other performance descriptors such as resolution^12,25^ and orthogonality are also calculated, but were not used in the present study). This step yields a set of results for all experimentally achievable configurations. In the final step, Pareto optimization is performed by removing all of the achievable results that are not optimal in terms of the specified quality descriptors. The complete set of optimal achievable results are plotted, typically in three dimensions with each axis representing one of the optimization objectives, to create a Pareto front where each point on the front represents the performance metrics corresponding to a set of experimental conditions. The experimental conditions corresponding to each point can be accessed, along with theoretical two- and three-dimensional “predicted” chromatograms (i.e., as 2D contour plots or three-dimensional (3D) surfaces).

The algorithm used by the optimization program is the same as described previously,^13^ but small improvements have been made since our most recent publication. These include using the retention time of the last eluting peak as a measure of the total analysis time and correcting the peak capacities in both dimensions based on the retention time of the last eluting peak. These modifications allow for some degree of gradient selection when orthogonality cannot be used for this purpose due to an insufficient number of analytes (e.g., when standards are used to represent a sample).

For this work, the most important modification of the optimization program is the inclusion of the numerical simulation to model injection band broadening in the second dimension, as an alternative to the theoretical injection equations. The numerical simulation model has been described in detail elsewhere.^17,18^ It is based on the Craig counter-current distribution model and quantifies the propagation of analyte mass though the column as a series of pseudo-counter-current distributions between two immiscible liquids using a discretized space and time grid.^26^ Plotting the analyte mass as a function of time at a distance equal to the column length gives the analyte mass elution profile. The model therefore enables the simulation of elution profiles of chromatographic peaks also under conditions where the sample solvent and mobile phase do not match. The model furthermore accounts for asymmetric injection profiles by using experimentally determined injection profiles obtained from 2D-LC modulation valves under full or partial sample loop filling conditions.^18^

The simulation model requires as inputs the retention parameters of the analytes, the column plate count, length, and void volume, the sample solvent and mobile phase composition at the time of injection, the sample loop volume and fill percentage, as well as the instrument dwell volume, flow rate, and gradient time. All of these values are also required for or calculated by the predictive optimization algorithm. Therefore, incorporating the simulation model into the optimization program is relatively straightforward. The only modification to the program involves obtaining retention times and peak variances from the output of the simulation model, as opposed to using theoretical relationships, as outlined above. An important consequence of this adaptation is that unlike the use of theoretical equations to estimate peak variance, where the peaks are assumed to be Gaussian, the simulation algorithm provides as output the elution profiles of the predicted peak shapes. These can then be used to create simulated 2D chromatograms that reflect actual peak shapes even if they are distorted due to mobile phase mismatch.

When included as a component of the Pareto optimization program, the simulation model must be run once per analyte for each combination of experimental variables. The time required to perform each calculation is highly dependent on the column efficiency, the retention time of the analyte, and the power of the computer performing the calculation. For the conditions considered in this paper, the algorithm requires approximately 1 h to simulate 100 conditions for 10 analytes on a Dell Optiplex 7460 computer with an Intel Core i7–8700 3.2 GHz processor (8 GB DDR4 ram).

Three algorithm sequences were used to obtain the necessary data sets for this study. In the first algorithm, the theoretical injection equation was used to estimate the ^2^D injection band broadening. The second algorithm used the numerical simulation model to predict the ^2^D elution profiles. The third algorithm used the theoretical injection equation to obtain a preliminary optimized Pareto front, followed by reoptimization of each point on the initial front using the numerical simulation model (the three algorithm sequences are illustrated schematically in Figure S1, while a synopsis of the third algorithm is presented in Figure 3). The input parameters used for all three algorithms are provided in Tables S1 and S2 in the SI. The optimization software can be downloaded from the link provided in the SI.

## Experimental Section

### Materials

Gallic acid (1), catechin (2), caffeic acid (3), vanillic acid (4), rutin (6), resveratrol (8), quercetin (9), naringenin (10), uracil, toluene, formic acid (FA), and HPLC grade methanol (MeOH) were purchased from Sigma-Aldrich (Darmstadt, Germany). Aspalathin (5) and isovitexin (7) were obtained from the Agricultural Research Council (E. Joubert, Stellenbosch, South Africa) and HPLC grade acetonitrile (ACN) was obtained from ROMIL (Cambridge, U.K.). Deionized water was obtained using a Milli-Q water purification system (Millipore, Milford, MA).

### Instrumentation

A Waters Acquity ultra-performance liquid chromatography (UPLC) system consisting of an autosampler, degasser, binary pump, column oven, and photodiode array (PDA) detector (500 nL flow cell) operated using Waters Empower software (Waters, Milford, MA) was used to measure the standard analyte constants (van Deemter coefficients and retention parameters) and column properties (porosity and resistance factors). The extra-column and dwell volumes for this system were determined as 20 and 145 μL, respectively.

For the LC × LC experiments, a Waters Acquity UPLC BSM pump and Agilent 1200 Micro Well Plate autosampler were used in ^1^D, and an Agilent 1200 column oven (with a built-in 6 μL preheater), a 1290 Infinity II binary pump (G7120A) and a 1290 Infinity II DAD detector (G7117A, 1 μL flow cell) were used in the ^2^D (Agilent Technologies, Waldbronn, Germany). A 2-position/8-port valve (Agilent) was used as the interface between the two dimensions. The valve was operated in the counter-current configuration using 20, 40, 60, 80, and 180 μL loops connected though zero-volume unions to obtain the required sample loop volumes (as specified in Table 1). An Agilent 1100 Isocratic pump provided a makeup flow, which was introduced between the outlet of the ^1^D column and the modulation valve using a T-piece. The measured dwell volumes were 13 and 55 μL for ^1^D and ^2^D, respectively (excluding the loop volume in the ^2^D). The extra-column variance of the ^2^D system was measured as 17 μL^2^ (after the valve) and 3.8 μL^2^ (after the column). All modules, including the valve, were controlled by OpenLab ChemStation Edition software (Agilent), except for the Acquity UPLC pump, which was controlled by Empower (Waters).

**Table 1:** Experimental Conditions Used for On-Line HILIC × RP-LC Analysis of Phenolic Standards^a^

point num.^b^	flow rate ^1^D (μL/min)	^1^D make-up flow (μL/min)	flow rate ^2^D (mL/min)	analysis time (min)^c^	^1^D gradient	sampling time (min)	^2^D gradient	loop volume (μL)	^1^D eluent fraction (μL)	^1^*n*′_c_	^2^*n*_c_	*n*_c,2D_	dil.
#1	17	68	2	41	1–25% in 55 min	0.75	1–55% in 0.56 min	80	12.75	32	82^d^	2579^d^	3.5^d^
											78^e^	2459^e^	5^e^
A (theor.)	17	153	2	41	1–25% in 55 min	1.1	1–55% in 0.47 min	230	18.7	27	105^d^	2810^d^	4.6^d^
											96^e^	2572^e^	7.7^e^
B (sim.)	17	68	2	41	1–25% in 55 min	0.65	1–55% in 0.84 min	70	11.05	34	75^d^	2510^d^	3.4^d^
											72^e^	2401^e^	4.7^e^
#2	17	153	2	41	1–25% in 55 min	0.75	1–55% in 0.52 min	160	12.75	32	79^e^	2499^e^	6.4^e^
#3	26	234	2	35.8	1–25% in 56.7 min	0.8	1–55% in 0.52 min	260	20.8	32	72^e^	2302^e^	6.9^e^
#4	29	116	2.5	34.5	1–25% in 57 min	0.65	1–55% in 0.49 min	120	18.85	35	71^e^	2514^e^	5.7^e^

a Abbreviations: ^1^D peak capacity corrected for undersampling (^1^*n*′_c_), ^2^D peak capacity (^2^*n*_c_), two-dimensional peak capacity (*n*_c,2D_), dilution factor (Dil.)

b Points #1, A, and B are indicated on the theoretical and simulated Pareto fronts in Figure 1; points #2–4 are specified on the simulated Pareto fronts presented in Figures S2 and S3 in the SI.

c based on predicted retention time of the last eluting compound in ^1^D.

d values calculated using the theoretical equation (eq 3).

e values calculated using the simulation model.

## Chromatographic Conditions

### Determination of Analyte Constants

Plate height and retention data for the standard analytes in HILIC and RP-LC were measured on an XBridge Amide column (150 × 2.1 mm, 1.7 μm, Waters) and a Zorbax Eclipse Plus C18 column (100 × 2.1 mm, 1.8 μm, Agilent), respectively. The mobile phases consisted of 0.1% FA in ACN (mobile phase A in HILIC and mobile phase B in RP-LC) and 0.1% FA in water (mobile phase B in HILIC and mobile phase A in RP-LC). Plate height measurements were performed isocratically at 50 °C with mobile phase compositions adjusted to obtain retention factors of >3 for all compounds (where possible). Retention data were obtained by performing three or four gradient analyses, with the gradient slopes between each analysis differing by a factor of 3. The *fminsearch* function in MATLAB was used to simultaneously solve for both *k*_100_ and *n* in the case of HILIC, and for both *k*_0_ and *S* in the case of RP-LC. HILIC retention values were measured using linear gradients of 1 to 30% B in 4, 12, 36, and 108 min, whereas RP-LC values were measured using linear gradients of 1 to 40% B in 8, 24, and 72 min. For both modes, a 0.3 mL/min flow rate was used, and the experiments were performed at 30 and 60 °C for HILIC and RP-LC, respectively. Ultraviolet (UV) data were recorded between 190 and 400 nm by using a 40 Hz acquisition rate.

### HILIC × RP-LC Experiments

^1^D separations were performed on an XBridge Amide column (150 × 1.0 mm, 1.7 μm, Waters) by using mobile phases consisting of (A) 0.1% FA in ACN and (B) 0.1% FA in water. In ^2^D, a Zorbax Eclipse Plus C18 column (50 × 3.0 mm, 1.8 μm, Agilent) was used with mobile phases consisting of (A) 0.1% FA in water and (B) 0.1% FA in ACN. A makeup flow of 0.1% FA in water was added to the ^1^D effluent at the outlet of the ^1^D column. The gradients, flow rates, and sampling times used for each analysis are specified in Table 1. ^1^D separations were performed at room temperature and ^2^D separations at 60 °C. UV detection was performed at 280 nm with an 80 Hz acquisition rate.

### Calculations

All calculations and figures were prepared using MATLAB R2018b and R2019b (Mathworks Inc., Natick, MA). The simulations for the large data set with the second algorithm sequence were run on virtual machines hosted by Amazon Web Services (Amazon, Seattle, WA).

## Results and Discussion

### Combining Elution Profile Simulation and Kinetic Optimization

The effective transfer of ^1^D effluent fractions to the ^2^D column under conditions that avoid excessive ^2^D band broadening is one of the main challenges in online LC × LC method development when passive or dilution modulation is used. This is especially true for systems where the mobile phases used in each of the dimensions differ significantly in terms of their elution strengths in the other, where injection band broadening can drastically affect method performance. It is therefore critically important for LC × LC method optimization protocols to accurately model ^2^D injection. We have previously found^13^ that existing theoretical models used for this purpose are inadequate for the prediction of online HILIC × RP-LC separations.

The aim of the present study was therefore to explore the option of incorporating an elution profile simulation algorithm into an LC × LC kinetic optimization protocol to more accurately model peak shapes for especially weakly retained analytes under strong solvent and/or volume overloading conditions. Such an approach would be useful in improving the accuracy of predictions used in LC × LC method development, while a further benefit would be that the simulated elution profiles could be used to construct predicted 3D surface plots, which would prove to be especially advantageous for method selection.

As a first step, we compared optimization results obtained using the simulation model to those calculated using the theoretical injection equation (refer to the Methodology Section for details). To do so, two separate Pareto optimizations were performed for the HILIC × RP-LC separation of phenolic compounds using the kinetic optimization program previously reported^13^ and briefly outlined in the Methodology Section. In the first optimization set, the theoretical injection equation was used, whereas the second used the simulation model. Ten standards comprising several common phenolic classes, for which plate height and retention parameters were determined experimentally in both HILIC and RP-LC (Table S1, SI), were used for this purpose. Other instrumental and chromatographic parameters used in these predictions are listed in Table S2. In order to minimize the computation time required for the elution profile simulations, the number of experimental parameter values considered in the optimization were limited, and fixed column dimensions were used (Table S2). The two Pareto fronts calculated using the injection equation (red) and the simulation model (blue) are presented in Figure 1, with each point on the figure representing a set of optimal experimental conditions. It is evident from Figure 1 that the simulation algorithm predicts lower achievable performance (higher total dilution and lower peak capacities) compared to the theoretical injection equation. The simulation model generally predicts more injection band broadening will occur for a given set of conditions.

**Figure 1:**
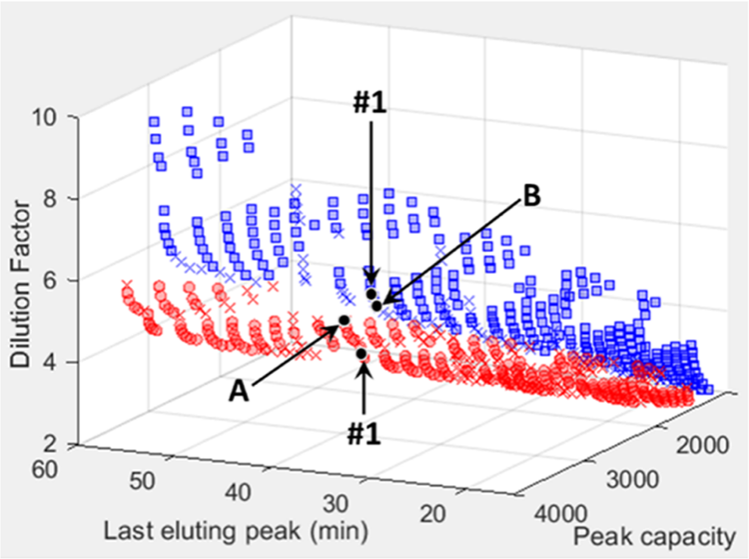
Comparison of the Pareto fronts for the HILIC × RP-LC separation of phenolic compounds calculated using the theoretical injection equation (red, circle open) and the simulation model (blue, open box). X’s represent conditions that appear on only one of the two fronts. Experimental parameters used for the optimization are provided in Tables S1 and S2 (SI), with a maximum ^2^D flow rate of 2 mL/min used for the fronts presented.

**Figure 2:**
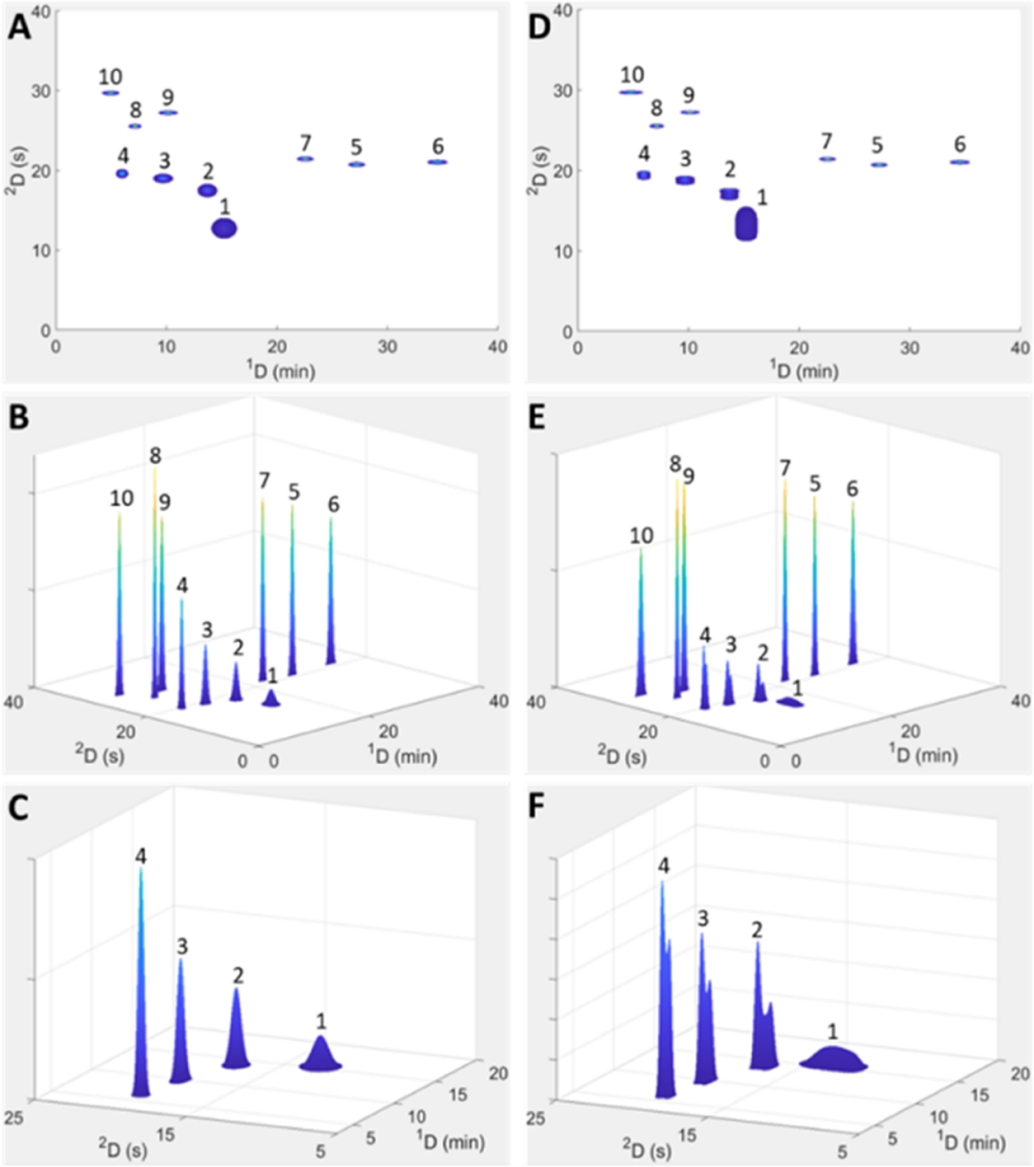
Comparison of the two- and three-dimensional HILIC × RP-LC contour plots of point #1 indicated on the Pareto fronts in Figure 1. Predicted contour plots were calculated using the injection equation model (A–C) and the simulation model (D–F) for the experimental conditions corresponding to point #1 specified in Table 1. (C, F) shows enlargements of the low retention time range in (B, E), respectively. Peak numbers correspond to the phenolic standards listed in the Materials Section.

To investigate the differences in predictions in more detail, we can compare the predicted contour plots of one of the optimal points occurring on both Pareto fronts in Figure 1 (marked point #1 on both fronts). The experimental conditions for this point are identical and are listed in Table 1. Briefly, these correspond to ^1^D and ^2^D flow rates of 17 μL/min and 2 mL/min, respectively, a sampling time of 0.75 min, a makeup flow of 68 μL/min, and a loop volume of 80 μL. In Figure 2, the predicted 2D and 3D contour plots of point #1, as calculated using the injection equation (Figure 2A,B) and simulation model (Figure 2D,E), are depicted, with corresponding enlargements of the 3D surface plots focused on the region where the weakly retained peaks appear in Figure 2C,F.

Comparing Figure 2A and 2D, it is evident that the predicted performance for the more retained compounds (peaks 5–10) is similar for both injection models. However, for the weakly retained compounds (peaks 1–4) that are most prone to injection band broadening, the simulation model predicts broader peaks, with the biggest difference being observed for the most weakly retained compound (gallic acid, peak 1). As a result of the larger predicted peak widths of compounds 1–4, the simulation model predicts a slightly lower ^2^D peak capacity (78, compared to 82 for the injection equation), which translates into an overall 2D peak capacity loss of 120. Perhaps more significantly, due to the larger peak widths predicted using the simulation model for the weakly retained analytes, as reflected in the much lower signal intensities for these peaks, the simulation model predicts a higher overall dilution factor (5 vs 3.5 for the injection equation, Table 1).

Although the predicted performance obtained using the theoretical injection equation and the simulation model differs, the sets of optimal experimental conditions appearing on the two Pareto fronts are fairly similar. In Figure 1, the sets of conditions that appear on both Pareto fronts are indicated with squares (□) and circles (○), respectively, while the points that exist on only one of the fronts are marked with crosses (X).

As an example of a point that exists only on the front calculated using the theoretical injection equation, it is informative to compare point A in Figure 1 to point #1, which occurs on both fronts. The experimental conditions for point A are listed in Table 1. The ^1^D gradient for both points is the same, but point A corresponds to a longer sampling time (1.1 min) and therefore a larger ^1^D fraction volume (18.7 μL compared to 12.75 μL for point #1). Under these conditions, a higher degree of injection band broadening is expected, and indeed the predicted ^2^D performance becomes too low when the simulation model is used (^2^*n*_c_ = 96), resulting in the removal of this point from the Pareto front. However, with the theoretical injection equation, the ^2^D performance is notably higher (^2^*n*_c_ = 105), and therefore, the point remains optimal on this front.

Despite a relatively small number of such differences, the majority of the optimal conditions on the two fronts in Figure 1 are similar. It can therefore be concluded that although the results of the theoretical injection equation and the simulation model differ quantitatively, the optimum conditions predicted by both models are fairly similar.

**Figure 3:**
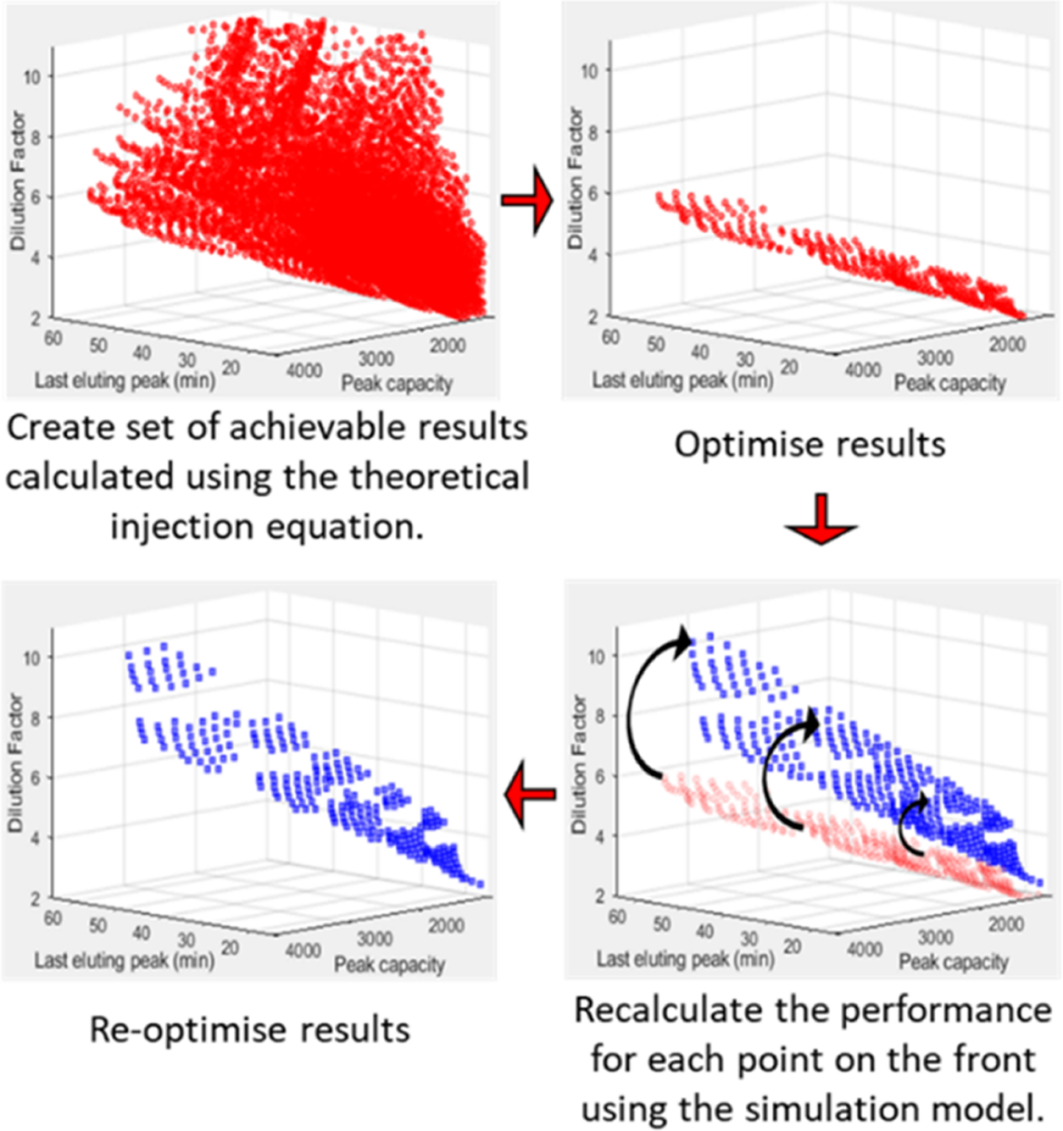
Flowchart showing the main steps in the proposed optimization methodology incorporating both a theoretical injection equation and the simulation model to limit the computational demand of simulation modeling. A detailed flowchart of the algorithm sequence is provided in Figure S1.

The simulations for the Pareto optimization in Figure 1 were performed using the computing power of Amazon Web Services, since even with the limited number of conditions evaluated, the simulations would require ∼324 h on a normal desktop computer. Performing a complete method optimization using the simulation model is thus not a currently viable option. Bearing in mind the similarities between the optimal points on the Pareto fronts obtained using the theoretical injection and simulation models, we propose performing an initial optimization step using a theoretical injection equation, and subsequently recalculating the performance of each point on the optimized Pareto front using the simulation model. This proposed methodology is summarized in Figure 3, with a detailed algorithm sequence diagram presented in Figure S1.

Because the injection equation and simulation model give similar optimum conditions (cf. Figure 1), the final Pareto front generated as output using this proposed methodology will only differ marginally from the one generated using only the simulation model. Significantly though, the number of simulations that must be performed is greatly reduced in this manner, and the computation time becomes reasonable.

The main drawback of this methodology is that some conditions that appear only on the Pareto front generated using the simulation model will be excluded from the final optimized front, even though they should strictly be included. These points generally correspond to conditions where a slightly lower ^1^D fraction volume (resulting from either a lower ^1^D flow rate or shorter sampling time) is transferred to the ^2^D column compared to the points close to them on the front. An example of such a point is point B in Figure 1, which occurs only on the simulated front and would therefore be excluded using the proposed protocol. The ^1^D gradient for this point is again identical to points #1 and A, but point B corresponds to a shorter sampling time (0.65 min) and smaller ^1^D eluent fraction volume (11.05 μL, Table 1). Accordingly, a lower degree of injection band broadening is expected, and the difference in ^2^D performance and total dilution predicted by the two models is considerably less. These conditions are excluded when the theoretical injection equation is used because the model predicts that the advantage of increasing the ^1^D flow rate (closer to optimum) or sampling time (higher ^2^*n*_*c*_) outweighs the loss in ^2^D peak capacity caused by more excessive ^2^D injection band broadening. This is not the case when the simulation model is used. Because the simulation model generally predicts a higher loss in the ^2^D peak capacity due to injection band broadening, the points corresponding to experimental conditions with smaller fraction volumes are preferred, and these conditions are included in the optimized front. While exclusion of these points on the final Pareto front is not ideal, it is not a fundamental problem since there are points in close vicinity on the front that provide comparable performance.

### Experimental Verification of the Advantages of Incorporating Elution Profile Information

**Figure 4:**
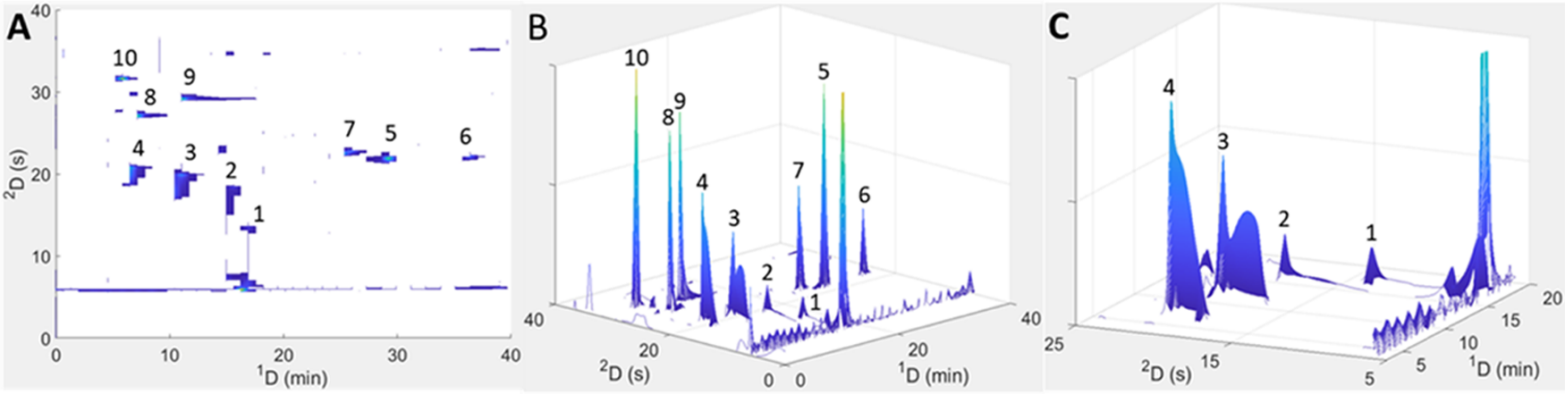
Experimental contour (A) and surface (B) plots recorded at 280 nm using the experimental conditions for point #1 specified in Table 1. (C) shows an enlargment of the low retention time range. For comparison to predicted contour plots, refer to Figure 2. Peak numbers correspond to the phenolic standards listed in the Materials Section.

One of the main advantages of the simulation model lies in its ability to predict the formation of deformed or split peaks caused by excessive injection band broadening. While the theoretical injection equation predicts an increase in ^2^D peak variance caused by band broadening, it provides no indication of the peak shape. Selecting LC × LC conditions based only on predicted peak variances—as opposed to elution profiles—can lead to unexpected and undesirable results. We demonstrate this using point #1 discussed in the previous section by comparing the predicted 2- and 3D contour plots calculated using the injection equation (Figure 2A–C) and simulation model (Figure 2D–F) with the experimental contour plots obtained using the conditions for this point (Table 1) in Figure 4. Note that the conditions corresponding to point #1 appear on the Pareto fronts obtained using both the theoretical equation and simulation models (Figure 1), due to the overall performance of this configuration calculated for all compounds. Looking at Figure 2A–C, it is evident that the theoretical injection equation predicts a high degree of injection band broadening for compounds weakly retained in the ^2^D (peaks 1–4). However, the peaks are still Gaussian, and apart from peak 1, the predicted separation might be considered acceptable. For the same set of conditions, the simulation model predicts the deformation of the weakly retained peaks, with peak splitting occurring for peaks 2–4 (Figure 2D–F). Split peaks are, of course, highly problematic from an identification and quantification point of view. In this instance, the additional peak shape information provided by the simulation model is especially useful, as it would allow an analyst particularly interested in the weakly retained analyte classes to conclude that these conditions may not be suitable for their application. Indeed, experimental data for this point shown in Figure 4 clearly confirm the presence of similarly deformed and split peaks for compounds 1–4. From these data, we can conclude that the information provided by the simulation model can greatly assist the chromatographer in choosing desirable LC × LC conditions.

**Figure 5:**
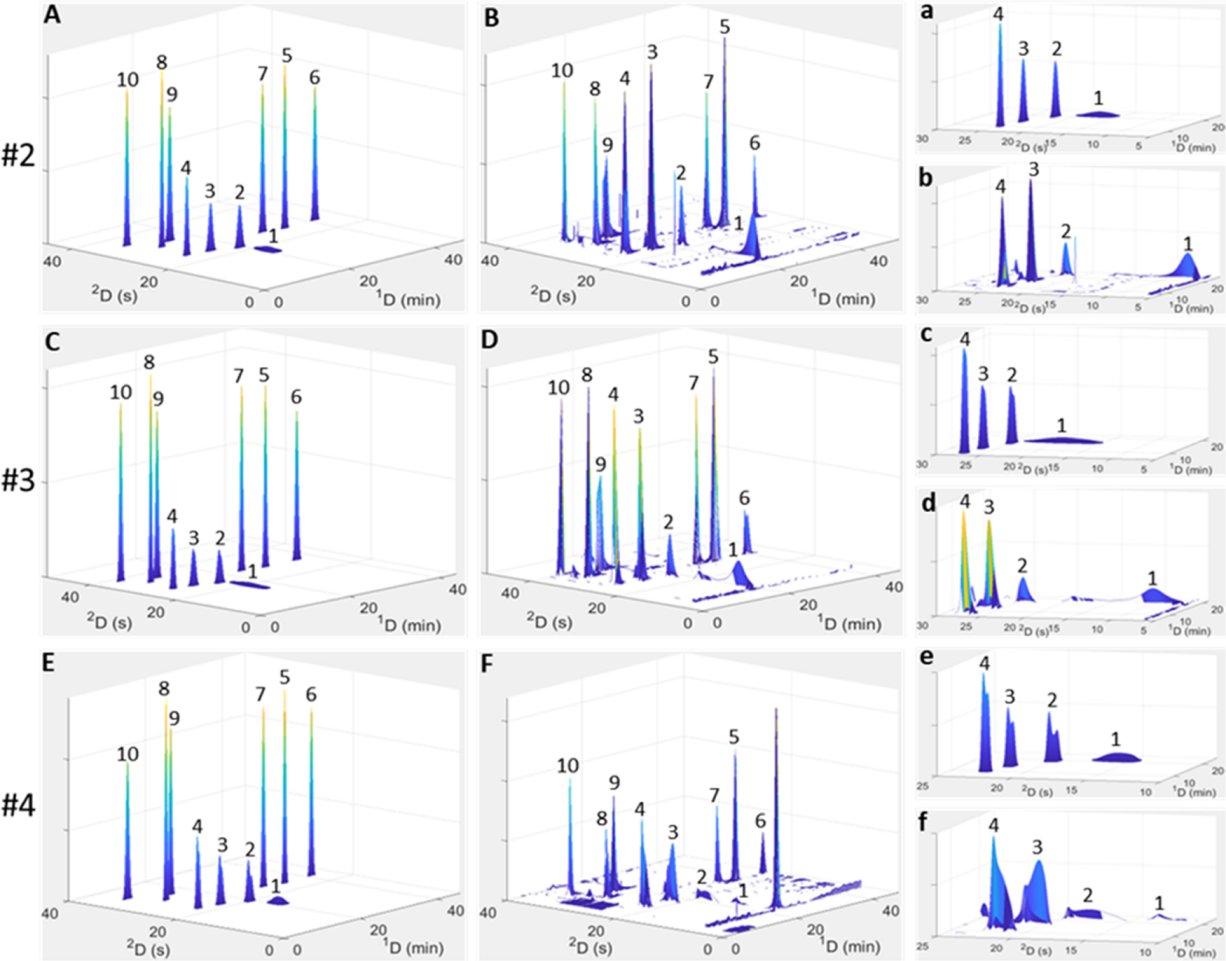
Comparison between predicted HILIC × RP-LC contour plots obtained using the simulation model (A, C, E) and the experimentally recorded data (B, D, F) for points #2–4 (Figures S2 and S3). For clarity, enlargements of the weakly retained ^2^D peaks in (A–F) are depicted in (a–f). Data were recorded at 280 nm using the experimental conditions listed for points #2–4 in Table 1. Peak numbers correspond to the phenolic standards listed in the Materials Section.

Figure 4 illustrates qualitative agreement between the simulated and experimental peak shapes, which is arguably the most important feature of the approach for LC × LC method optimization. While the validity of both the predictive kinetic optimization and simulation models have been demonstrated in previous work,^13,17,18^ the accuracy of their combined use in LC × LC method optimization as proposed here was also assessed experimentally in the present work. For this purpose, three sets of conditions present on the Pareto fronts obtained using the proposed approach of an initial optimization using the theoretical injection equation, followed by simulation of the optimal points and reoptimization, were used. The Pareto fronts used for this purpose were obtained using the parameter values listed in Tables S1 and S2 with modulator loop volumes between 80 and 260 μL and maximum ^2^D flows of 2 and 2.5 mL/min, and are presented in Figures S2 and S3, respectively. The points on these fronts used for experimental verification are labeled #2–4 in these figures. The experimental conditions for each point are summarized in Table 1. For visual comparison, the predicted and experimental 3D contour plots of the three points are depicted in Figure 5. In addition, Tables S3 and S4 show a comparison of the simulated and experimentally measured standard deviations for each of the analytes in the ^2^D measured at 50% height and using the second moment, respectively. Overall, the agreement between experimental and predicted 2D chromatograms in Figure 5 is sufficient for method selection or optimization purposes. It is clear from Table S3, however, that experimental peak widths are in almost all cases larger than those predicted by the simulation. The only exception is for peak 1 (gallic acid), where extensive breakthrough is observed in the experimental data. The severely distorted peak shape for this compound results in unrealistically small experimental half-height peak widths, as these are measured for the completely unretained “breakthrough” peak. In this case, the second moment provides a better indication of peak width (Table S4), with the experimental standard deviation of peak 1 being larger than predicted by the simulation. The remaining weakly retained compounds (peaks 2–4) show larger discrepancies compared to the predicted data than the more strongly retained ones.

One possible contributing factor to this deviation is additional extra-column band broadening occurring between the modulation valve and the ^2^D column. In the optimization program, only extra-column band broadening after the ^2^D column is taken into account, since focusing at the inlet of the column should nullify broadening before the column. However, weakly retained compounds that are subject to injection band broadening are not effectively focused, and therefore, extra-column band broadening before the column is expected to result in larger peak widths for such compounds. On the instrument used for experimental verification, the extra-column variance between the modulation valve and ^2^D column was measured as 13 μL^2^. This likely explains why the difference between the simulated and experimental peak widths is generally larger for the weakly retained compounds (peaks 2–4) than for the highly retained compounds (peaks 5–10).

A further contributing factor to the increased experimental peak variance may be the formation of radial temperature gradients caused by frictional heating.^27^ The plate height data used to predict peak widths were measured on a 2.1 mm internal diameter (i.d.) column, while the experimental analyses were performed using a 3 mm i.d. column at high pressures and flow rates. Under the latter conditions, frictional heating is expected to be more pronounced, and even in the still-air oven used here, a residual radial temperature gradient would negatively impact column performance. This effect is not modeled by the plate height data measured on the 2.1 mm i.d. column.

To investigate the potential influence of a radial temperature gradient in the ^2^D, the ^2^D column was placed in an insulating polystyrene housing inside the column oven (all ^2^D analyses were performed at 60 °C). Based on three separations repeated with and without the polystyrene housing, an average peak width decrease of 8.6% was measured with the polystyrene insulation. These data support the hypothesis that frictional heating on the 3 mm inner diameter column contributes to the observed differences between the simulated and experimental peak variances (indeed, for the more strongly retained compounds (5–10), an 8.6% decrease in peak width would bring the experimental values very close to predicted ones). It is important to note that, despite the quantitative discrepancies between predicted and measured peak variances, there is a high degree of correlation between the simulated and experimental contour plots. Importantly, peaks that appear split or deformed in the simulated contour plots are also deformed in the experimental data, and where the simulation predicts Gaussian peaks, this is affirmed by the experimental data. This confirms the ability of the simulation model to accurately predict the formation of split or deformed peaks due to injection band broadening. This information is crucial in selecting experimental conditions during method development.

## Conclusions

A methodology was developed to incorporate a chromatographic simulation model, capable of predicting peak elution profiles, into theoretical multiparameter 2D-LC method optimization software. Performing complete LC × LC optimization using the simulation model is not a realistic possibility due to the computational time required per simulation. However, by performing a preliminary optimization using a suitable theoretical injection equation to estimate injection band broadening and only applying the simulation model to the points on the preliminary optimal front obtained in this manner, the number of simulations that need to be performed for the final optimization is reduced to a feasible number. We showed that the optimized conditions obtained in this manner are comparable to results generated through a full optimization using only the simulation model, while the required computation time is greatly reduced.

Application of the simulation model as part of a method optimization algorithm was demonstrated for the HILIC × RP-LC separation of phenolic compounds. It was shown that the simulation model can accurately predict the formation of split or deformed ^2^D peaks for a variety of experimental conditions during the method development stage. This capability of the proposed approach is invaluable in preventing the selection of experimental conditions that will result in undesirable separation based on averaged performance metrics. The possibility of evaluating predicted two- and three-dimensional contour plots constructed from the elution profiles of the simulations is therefore a crucial benefit when it comes to selecting suitable 2D-LC conditions.

Experimental verification of the developed approach was performed by comparing the ^2^D peak variances and elution profiles for phenolic compounds analyzed by HILIC × RP-LC to the simulated contour plots (i.e., 2D chromatograms). Some discrepancies between the predicted and experimentally measured peak variances were noted, especially for weakly retained compounds in the ^2^D. However, evaluation of the elution profiles showed that the experimental data displayed deformed peaks for the same compounds and conditions as predicted by the simulated contour plots.

We believe that the superior elution profile information provided by the simulation model compared with other theoretical models used to account for ^2^D injection band broadening is an exceptionally useful feature in 2D-LC method development. The reported protocol can be employed to great benefit as part of the method optimization process, especially in circumstances where sample solvent mismatch and sample volume overloading are likely to occur in the ^2^D, and where thorough optimization through experimentation is not feasible due to time and resource constraints.

## Supporting Information Available

Analyte properties and parameter ranges used in optimization; algorithm sequences; Pareto fronts used for experimental verification; comparison of simulated and experimental 2D peak widths; and method optimization app (PDF)
