# Supplementary material for: Improving the Accuracy of Predictive 2D-LC Optimization Strategies: Incorporation of Simulated Elution Profiles to Account for Injection Band Broadening in Online Comprehensive Two-Dimensional Liquid Chromatography
Source: Anal Chem. 2024 Apr 9;96(16):6398–407. doi: 10.1021/acs.analchem.4c00491 (PMC11044102; doi:10.1021/acs.analchem.4c00491)
Supplement: Supplementary file 1 — ac4c00491_si_001.pdf [file ac4c00491_si_001.pdf]

Supporting Information

Improving the accuracy of predictive 2D-LC optimization strategies: Incorporation of simulated elution profiles to account for injection band broadening in on-line comprehensive two-dimensional liquid chromatography

Magriet Muller[1], Tyler Brau[2], Thomas Lauer[2], Dwight Stoll[2] and André de Villiers[1]*

[1]Department of Chemistry and Polymer Science, University of Stellenbosch, Private Bag X1, Matieland, 7602, South Africa

[2]Department of Chemistry, Gustavus Adolphus College, 800 West College Avenue, Saint Peter, 56082, MN, United States

*Corresponding author. Tel.: +27 21 808 3351; fax +27 21 808 3360. E-mail: ajdevill@sun.ac.za

Table of Content

Table S1. Parameters used in the optimization protocol. S2

Table S2. Parameter ranges used in the construction of the Pareto curves S2

Figure S1. The three algorithm sequences used for Pareto-optimization in this work. S3

Figure S2. Pareto front used for experimental verification (maximum ^{2}D flow 2 mL/min). S4

Figure S3. Pareto front used for experimental verification (maximum ^{2}D flow 2 mL/min). S4

Table S3. Comparison of simulated and experimental (half height) ^{2}D peak standard deviations. S5

Table S4. Comparison of simulated and experimental (2nd moment ^{2}D peak standard deviations. S5

Method optimization app S6

Table S1. Analyte plate height and retention parameters used in the optimization protocol.

	RP-LC					HILIC				
	Reduced van Deemter			Retention parameters		Reduced van Deemter			Retention parameters	
Name	***a***	***b***	***c***	**ln(k$_0$)**	**S**	***a***	***b***	***c***	**ln(k$_{100}$)**	**n**
Gallic acid	4.69	48.3	3.81	0.52	5.61	2.84	1.15	0.15	-2.73	1.30
Catechin	17.5	5.01	0.27	2.95	23.2	1.09	1.76	0.17	-2.65	1.20
Cafeic acid	24.6	3.37	1.25	2.96	18.7	0.75	2.70	0.15	-2.97	1.07
Vanillic acid	2.36	2.13	0.99	2.94	17.1	0.85	2.69	0.14	-3.86	1.00
Aspalathin	1.26	1.90	1.02	5.17	27.9	0.98	1.96	0.12	-3.36	2.09
Rutin	2.64	2.66	1.57	5.79	30.3	1.12	2.36	0.12	-3.77	2.61
Iso-Vitexin	0.01	5.22	0.58	5.70	28.7	1.02	2.41	0.13	-2.45	1.54
Resveratrol	0.74	1.67	0.75	5.09	18.2	0.93	2.35	0.14	-4.21	1.20
Quercetin	12.5	5.98	1.09	5.91	19.1	2.22	2.02	0.00	-3.12	1.14
Naringenin	12.5	5.98	1.09	5.90	16.4	2.22	2.02	0.00	-9.01	2.09

Table S2. Column dimensions and experimental parameter ranges used in the construction of the Pareto curves.

	**Name**	**Values (^{1}D; ^{2}D)**	**Interval**	**Permutations**
Variables	Column length	150 mm; 50 mm		
	Column diameter	1 mm; 3 mm		
	Particle size	1.7 µm; 1.8 µm		
	Total time	30-60 min	30 min	2
	Column flow resistance	1000; 1000		
	Column total porosity	0.626; 0.611		
	Temperature	30°C; 60°C		
	Solvent A	ACN; H_2O		
	Solvent B	H_2O; ACN		
	Initial mobile phase composition	1% B; 1% B		
	Final mobile phase composition	15-35% B; 35-55% B	10%	3; 3
	Dwell volume (excluding loop volume)	13 µL; 55 µL		
	Flow rate in ^{1}D	5-32 µL/min	3 µL/min	10
	Flow rate in ^{2}D	2-2.5 mL/min	0.5 mL/min	2
	Sampling time	0.3-0.8 min<br>0.9-1.2 min	0.05 min<br>0.1 min	15
	Column volumes for re-equilibration	[a]NA; 1.1		
	^{1}D injection volume (% $^1V_0$)	2.5%		
	^{1}D sample solvent	5% B		
	Dilution of ^{1}D eluent	3, 5, 10, 15, 20, 30 times		6
Restrictions	Sample loop fill %	[a]NA; 80%		
	Max. pressure	400 bar; 1000 bar		
	Max. available loop volume	260 µL		
	Total permutations			32400

[a]not applicable.

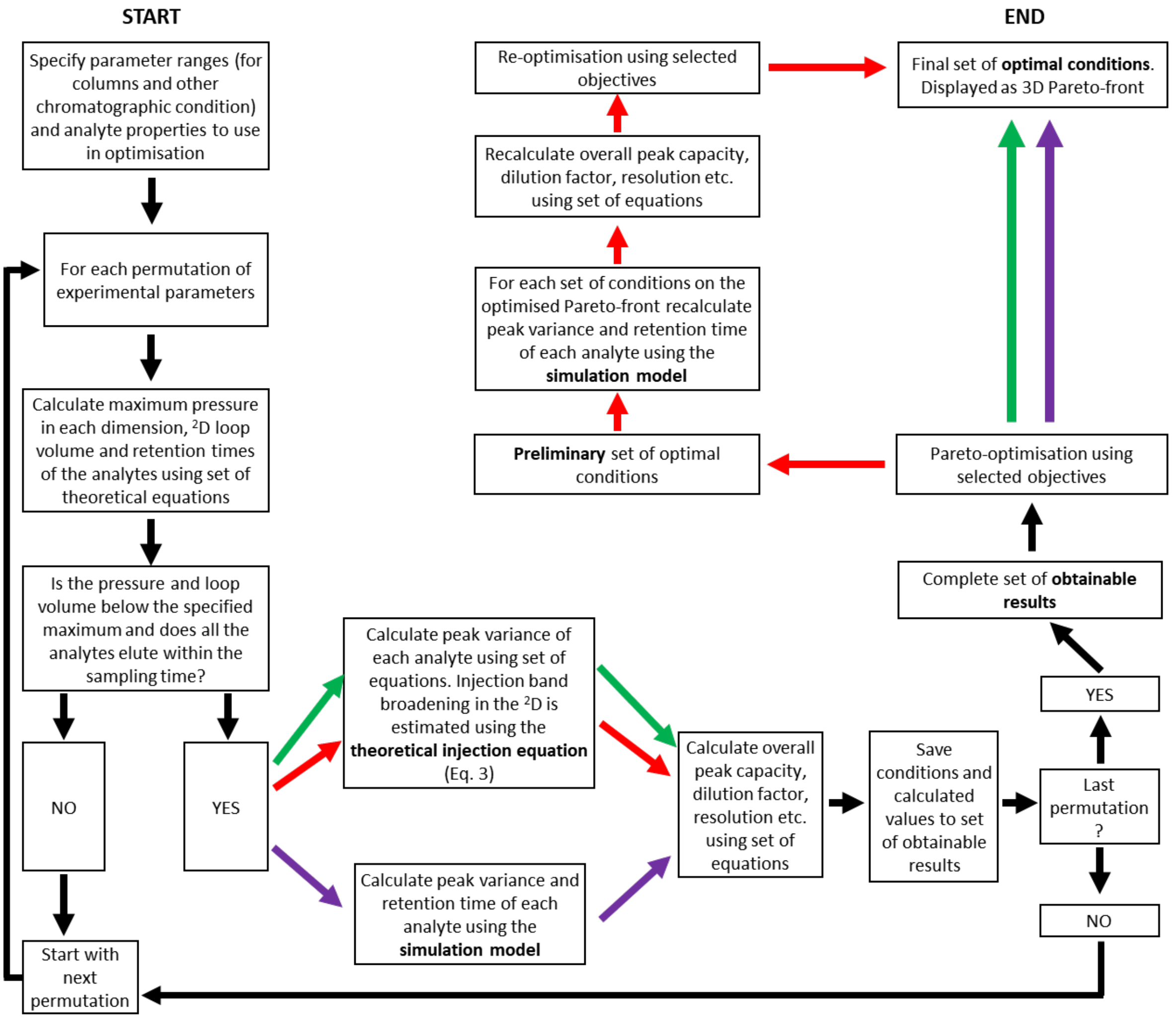

Figure S1. The three algorithm sequences used for Pareto-optimization in this work. Black arrows indicate steps followed by all three algorithm sequences. Green arrows represent the steps followed by the first algorithm that uses the theoretical injection equation to estimate injection band broadening, and the purple arrows the steps followed by the second algorithm that uses the simulation model to predict injection band broadening. The red arrows represent the third algorithm where the theoretical injection equation is used to calculate the initial Pareto front, followed by recalculation of each point on the front using the simulation model.

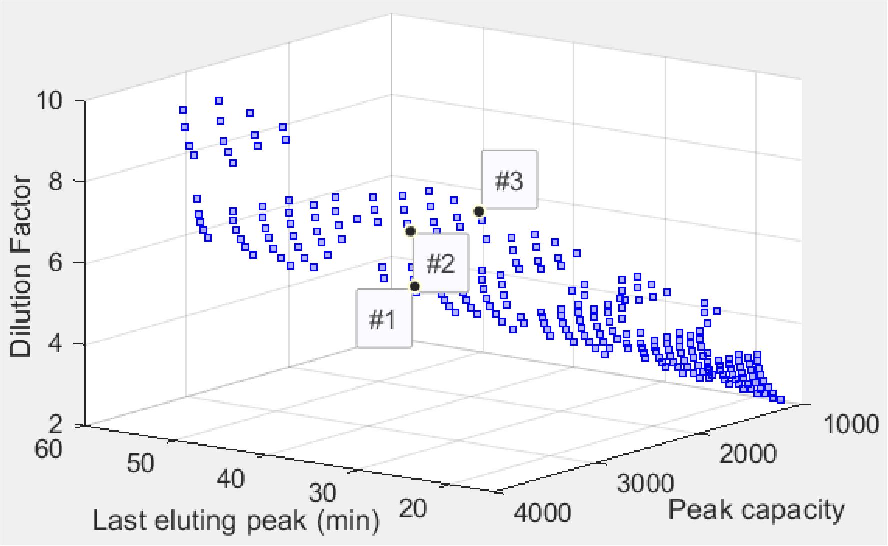

Figure S2. Pareto front used for experimental verification of the proposed method optimization approach. The front was generated using the parameters specified in **Table S2**, with a maximum ^{2}D flow rate of 2 mL/min. Conditions of the three points (marked #1-3) used for experimental verification as well as their performance metrics are provided in **Table 1**.

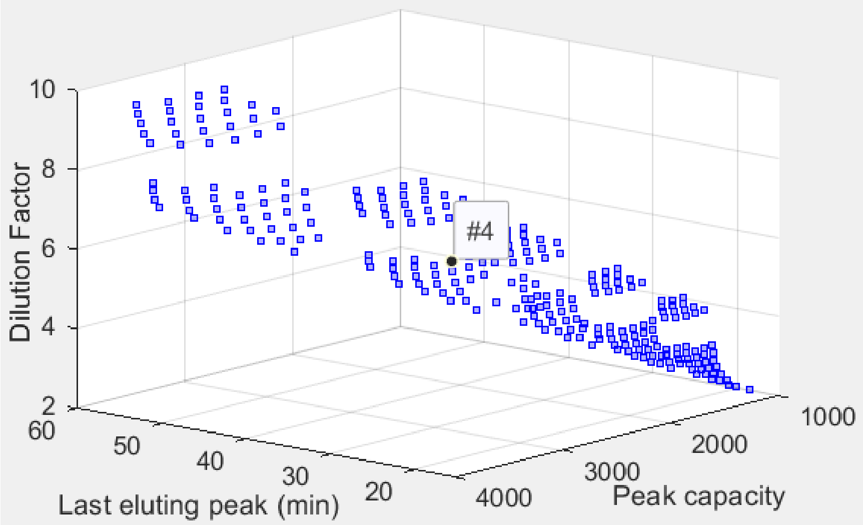

Figure S3. Pareto front used for experimental verification of the proposed method optimization approach. The front was generated using the parameters specified in **Table S2**, with a maximum ^{2}D flow rate of 2.5 mL/min. Conditions of the point (marked #4) used for experimental verification are provided in **Table 1**.

Table S3. Comparison[a] of simulated[b] and experimental[c] ^{2}D peak standard deviations for three sets of experimental conditions, points #2-4 specified in **Table 1**.

Point #[d]	Peak[e]:	1	2	3	4	5	6	7	8	9	10
**#2**	Simulated σ (s)	1.15	0.16	0.15	0.15	0.10	0.10	0.10	0.10	0.09	0.10
	Experimental σ (s)	0.80	0.31	0.27	0.23	0.10	0.10	0.10	0.11	0.11	0.11
	**% difference**	**44**	**-49**	**-43**	**-34**	**-2.0**	**-1.0**	**-3.0**	**-7.3**	**-10**	**-11**
**#3**	Simulated σ (s)	0.74	0.26	0.20	0.19	0.10	0.10	0.10	0.10	0.10	0.10
	Experimental σ (s)	1.16	0.49	0.36	0.32	0.10	0.10	0.10	0.12	0.11	0.11
	**% difference**	**-36**	**-46**	**-46**	**-40**	**-1.0**	**-4.2**	**-2.0**	**-10**	**-12**	**-8**
**#4**	Simulated σ (s)	2.08	0.24	0.22	0.22	0.10	0.10	0.10	0.10	0.10	0.10
	Experimental σ (s)	0.28	1.32	0.68	0.41	0.13	0.11	0.14	0.14	0.12	0.11
	**% difference**	**648**	**-82**	**-68**	**-46**	**-25**	**-14**	**-31**	**-24**	**-17**	**-15**

[a]percentage difference calculated as: 100×(Sim-Exp)÷Exp.
[b]calculated from 2nd moments of simulated peaks.
[c]measured at half height (2.354 σ).
[d]Points #2-4 are indicated on the Pareto fronts presented in **Figures S2** and **S3**.
[e]Peak numbers correspond to the phenolic standards listed in Materials.

Table S4. Comparison[a] of simulated[b] and experimental[c] ^{2}D peak standard deviations for three sets of experimental conditions, points #2-4 specified in **Table 1**.

Point #[d]	Peak[e]:	1	2	3	4	5	6	7	8	9	10
**#2**	Simulated σ (s)	1.15	0.16	0.15	0.15	0.10	0.10	0.10	0.10	0.09	0.10
	Experimental σ (s)	2.53	0.27	0.23	0.25	0.14	0.13	0.12	0.13	0.14	0.14
	**% difference**	**-55**	**-41**	**-35**	**-38**	**-32**	**-25**	**-22**	**-23**	**-32**	**-29**
**#3**	Simulated σ (s)	0.74	0.26	0.20	0.19	0.10	0.10	0.10	0.10	0.10	0.10
	Experimental σ (s)	4.60	0.44	0.35	0.34	0.13	0.11	0.13	0.15	0.13	0.14
	**% difference**	**-84**	**-41**	**-44**	**-45**	**-23**	**-5.3**	**-23**	**-30**	**-26**	**-29**
**#4**	Simulated σ (s)	2.08	0.24	0.22	0.22	0.10	0.10	0.10	0.10	0.10	0.10
	Experimental σ (s)	2.38	2.90	2.46	0.61	0.19	0.14	0.13	0.15	0.12	0.12
	**% difference**	**-0.13**	**-92**	**-91**	**-63**	**-48**	**-30**	**-24**	**-34**	**-22**	**-17**

[a]percentage difference calculated as: 100×(Sim-Exp)÷Exp.
[b]calculated from 2nd moments of simulated peaks.
[c]calculated from 2nd moments of experimental peaks.
[d]Points #2-4 are indicated on the Pareto fronts presented in **Figures S2** and **S3**.
[e]Peak numbers correspond to the phenolic standards listed in Materials.

Method optimization app

An app version of the optimization program used can be accessed at https://www0.sun.ac.za/chemistry/advanced-separations/. This is a prototype research tool, which may require modification of individual scripts for specific applications.
